# Genetic Analysis of the Brown Bear Sub-Population in the Pindos Mountain, Central Greece: Insights into Population Status and Conservation

**DOI:** 10.3390/ani14233530

**Published:** 2024-12-06

**Authors:** Tzoulia-Maria Tsalazidou-Founta, Nikoleta Karaiskou, Yorgos Mertzanis, Ioannis Sofos, Spyros Psaroudas, Dimitrios Vavylis, Vaios Koutis, Vassiliki Spyrou, Athanasios Tragos, Yannis Tsaknakis, Antonia Touloudi, Alexios Giannakopoulos, Dimitrios Chatzopoulos, Charalambos Billinis, Maria Satra

**Affiliations:** 1 Faculty of Veterinary Medicine, University of Thessaly, 43100 Karditsa, Greece; tmtsalazidou@uth.gr (T.-M.T.-F.); alexiosg@yahoo.gr (A.G.); billinis@vet.uth.gr (C.B.); 2 Department of Genetics, Development & Molecular Biology, Faculty of Science, School of Biology, Aristotle University of Thessaloniki, 54124 Thessaloniki, Greece; nikolbio@bio.auth.gr; 3 Callisto Wildlife and Nature Conservation Society, 54640 Thessaloniki, Greece; mertzanis@callisto.gr (Y.M.); spyros@callisto.gr (S.P.); athantra2015@gmail.com (A.T.); gtsaknakis69@gmail.com (Y.T.); 4 Faculty of Public and One Health, University of Thessaly, 43100 Karditsa, Greece; gs.sofos@gmail.com (I.S.); atoul@uth.gr (A.T.); dchatzopoulos@uth.gr (D.C.); 5 Trikala Development Agency, 42200 Kalampaka, Greece; vavylis@kenakap.gr (D.V.); bkoutis@kenakap.gr (V.K.); 6 Faculty of Animal Science, University of Thessaly, 41222 Larissa, Greece; vasilikispyrou@uth.gr

**Keywords:** *Ursus arctos*, microsatellite loci, genetics, conservation, population structure

## Abstract

Habitat fragmentation poses a significant threat to the existence and reproduction of large carnivores, such as brown bears, as it affects the genetic connectivity of populations and, consequently, their long-term viability. Understanding the genetic makeup and dispersal patterns in areas where brown bears live is crucial for developing effective conservation plans and promoting human-brown bear coexistence. In this study, 214 hair samples were collected non-invasively from brown bears and were genetically analyzed using fifteen specific microsatellite loci to shed light on the genetic status and demography of a sub-population residing in Central Greece (Trikala-Meteora area). The broader Central and South Pindos regions have not been studied for over a decade; however, high levels of genetic diversity, a lack of heterozygosity deficiency, and no signs of inbreeding, along with an estimated effective population size (Ne = 99), confirm the good conservation status of this sub-population. Moreover, the existence of a natural and apparently functional corridor in Central Pindos was demonstrated by bear movements between the western and eastern parts of the project area, enhancing the sustainability of the population. These findings will contribute to future efforts to conserve natural corridors that enable functional connectivity among large areas of brown bear territories in Greece.

## Simple Summary

Fragmented habitats threaten animals by reducing genetic diversity. It is essential to understand the genetic composition and movement patterns of brown bears for effective conservation strategies and fostering coexistence with humans. This study analyzed 214 hair samples collected non-invasively from brown bears in the Trikala-Meteora area of Central Greece, revealing the genetic status and demographics of a local sub-population. Although the broader Central and South Pindos regions have not been examined in over ten years, findings indicate high genetic diversity, no signs of inbreeding, and an estimated effective population size of 99, suggesting a healthy conservation status. Additionally, a natural corridor facilitating bear movement between the western and eastern sections of the study area supports the population’s sustainability. These results will aid in future conservation efforts aimed at maintaining natural corridors for brown bear habitats in Greece.

## 1. Introduction

The brown bear (*Ursus arctos*) is one of the most widespread large carnivores in Greece, with its distribution covering large areas, mainly the Rodopi mountain range, the Prespes region, and the Pindos mountain range, as well as the intervening mountainous and forested areas. It appears that the brown bear population size in Greece is generally stable, showing regionally positive trends and characterized by a high rate of recolonization of former or new ranges [1]. In accordance with previous studies based on genetic data (mtDNA and microsatellite loci), the population comprises three highly differentiated genetic clusters, consistent with their geographical distribution in the Pindos, Prespes, and Rodopi mountain ranges [2]. Nevertheless, the species is still considered “endangered” (Red Data Book of the mammals of Greece) [3], while human-induced pressures and threats, such as poaching, road-related mortality, and habitat fragmentation due to large infrastructure development, continue to affect its survival potential [4,5]. For the orientation and adjustment of conservation plans and actions (such as the National Bear Action Plan) [5] regarding brown bears in Greece, it is important to consider the dynamics of the brown bear population. Therefore, to acquire the most accurate genetic information possible about the brown bear sub-populations distributed across the different Hellenic mountainous regions, several LIFE projects have been implemented over the last two decades.

Estimating population size and genetic diversity is crucial for the conservation and effective management of endangered species, such as the brown bear in Greece. Understanding these factors contributes to assessing the genetic status of sub-populations, making informed decisions about habitat protection, and implementing effective management strategies. However, monitoring bear populations can be particularly challenging due to their cryptic and solitary nature, as well as their occurrence at relatively low densities in the field. A large number of recent studies focusing on the ecology, demography, population genetics, and phylogeography of brown bears have incorporated non-invasive genetic sampling (NIGS) techniques [2,6,7,8,9,10,11,12]. This approach is especially valuable for large carnivores, as the collection of biological material from these animals can be demanding. Traditional methods involving the capture or handling of wild animals often present significant difficulties and require specialized skills and equipment. In recent years, scientists have increasingly utilized NIGS methods that do not rely on live trapping or individual marking. For example, traps designed for collecting hair samples for subsequent genetic analysis have proven to be highly effective [13,14,15]. This innovative approach not only minimizes stress and potential harm to the bears but also allows researchers to gather essential genetic data without the challenges associated with direct handling. NIGS is widely recognized as a reliable alternative sampling method, not only for genetic studies of animal species but also for estimating their population sizes [16,17,18,19,20]. By utilizing NIGS, researchers can obtain important insights into population structure, genetic diversity, and overall population dynamics, which are essential for developing targeted conservation strategies and ensuring the long-term survival of endangered species, such as the brown bear in Greece.

In this study, we utilized non-invasive genetic sampling (NIGS) methods to focus on the genetic analysis of microsatellite loci from hair samples collected from brown bears inhabiting the Trikala-Meteora area, located in the western region of Thessaly in Central Greece. This sub-population is a critical component of the overall brown bear population in Greece, serving as a vital source for bear recolonization to the east and south [21]. The Central and South Pindos regions have not been investigated for over a decade regarding the genetic status of their brown bear population [22]. This lack of recent research highlights the need for updated genetic assessments to inform conservation strategies. Furthermore, at a broader geographical scale, this sub-population plays a key role in maintaining gene flow through a significant mega-corridor that connects the Trikala-Meteora area with the Amyntaio region, part of the Prespes bear sub-population range [22]. Therefore, the primary objective of this study was to gain essential insights into the population status of the brown bears in this region by assessing genetic diversity, detecting potential bottlenecks, and identifying signs of inbreeding. Understanding these factors is crucial for effective conservation management and ensuring the long-term survival of brown bears in the Trikala-Meteora project area. By elucidating the genetic dynamics of this sub-population, we aim to provide valuable information that can guide future conservation efforts and promote the resilience of these majestic animals in a rapidly changing environment.

## 2. Materials and Methods

### 2.1. Sampling

**Figure 1:**
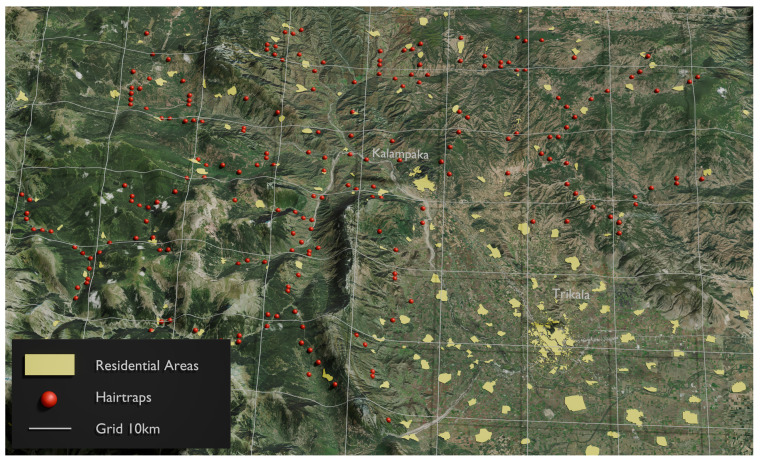
The network of 217 hair traps (red dots) in the Trikala-Meteora study area.

Hair traps on power poles are one of the sampling procedures that have been proposed for non-invasive genetic studies of brown bears [15]. This sampling method is based on the observation that bears use the wooden power poles for marking and rubbing, since they are probably attracted by the creosote embedded in the poles as a preservative prior to installation [15,23]. Marking behavior aims to (a) declare their presence during the reproduction period and (b) clear out parasites due to the presence of creosote. A hair trap is a single-stranded barbed wire 2.5–3 m long, hammered around the pole, forming a helix of metal rings. Depending on topography, poles are usually placed 50 to 100 m apart, and vegetation is cleared 5 m from each side of the power pole line [24]. A sampling network of 217 barbed wire-fitted poles (hair traps) was installed in the broader area of Trikala-Meteora (Figure 1) for systematically collecting the hair samples for the genetic analysis, and it was revisited monthly from May to August 2022. The selection of these specific poles were made at the beginning of the study, after inspection of the local power pole network for recent bear signs such as claw marks, hairs, or mud that indicate the “active” poles. A tuft of hair found on one barb of the barbed wire on a given power pole was considered as one sample. The samples were placed in uniquely numbered paper envelopes, without contact with human skin, labeled with the exact location, coordinates, and date of collection. After collection, the paper envelopes with hair samples were placed in zip-lock bags containing silica gel desiccant and stored at −20 °C until being analyzed in the laboratory. In total, 347 samples were collected and used in this study.

### 2.2. DNA Extraction from Hair Samples

**Figure 2:**
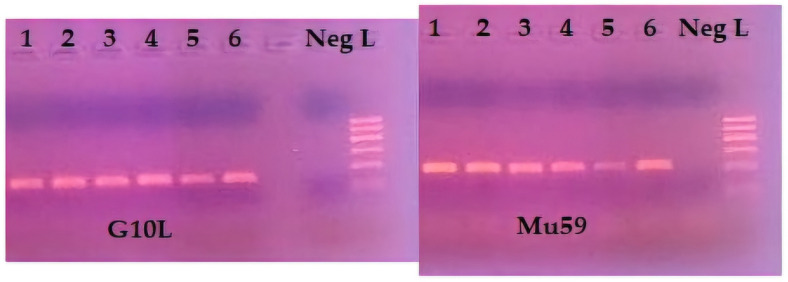
Gel images after PCR amplification for samples 1–6 regarding the microsatellite loci G10L, Mu59. Neg.: negative control, L: 100 bp DNA Ladder.

For the genetic analysis in this study, 2–12 hair roots per sample were used for DNA extraction. Each tuft of hair on a set of barbs was considered a sample. Using a stereoscope, hair roots were initially cut and transferred to a 1.5 mL tube. DNA was extracted from hair roots using the “QIAamp DNA Mini Kit” (Qiagen, Hilden, Germany), following the manufacturer’s instructions. The low quantity of obtained DNA does not allow direct testing of successful extraction in agarose gel. Thus, successful DNA extraction was evaluated through PCR amplification. To evaluate the amplification of the fifteen specific microsatellite loci, all PCR products were electrophoresed using 2% agarose gel (Figure 2).

### 2.3. PCR Amplification of Microsatellite Loci

Fifteen specific brown bear microsatellite loci were studied in the present study (Table 1). Thermal cycling was performed using a MJ Research (Peltier Thermal Cycler) PTC-200 thermocycler with 96-well “gold” blocks and QIAamplifier 96 (230 V) (Qiagen, Hilden, Germany). Specifically, PCR amplification of the microsatellite loci was accomplished using the following conditions: initial denaturation at 95 °C for 15 min, 45 cycles of strand denaturation at 94 °C for 30 s, annealing at 40–60 °C for 45 s, and elongation at 72 °C for 1 min. Final elongation was achieved at 72 °C for 10 min. Amplifications were performed with a reaction volume of 10 μL, containing 1 μL of 10× Reaction Buffer, 0.1 μL of 10× BSA, 0.25 mM dNTPs, 1p/μL of each primer, and approximately 50 ng of template DNA. Moreover, 0.4 units of Taq polymerase and 2 mM MgCl_2_ were used. Visualization of the PCR products was achieved by electrophoresis in 2% agarose gel. In order to identify the exact length of each microsatellite locus that was successfully amplified, capillary electrophoresis was performed through the QIAxcel Advanced system.

PCR assays were performed twice for all samples, following the Adams and Waits (2007) method [31], and each assay always included a negative control (blank) to decrease the probability of genotyping errors. Subsequently, capillary electrophoresis was performed for each successful PCR product only when the negative control (blank) was clear (absence of DNA product). Some samples were identified as mixed samples (with hair from >1 bear) by evidence of ≥3 alleles at ≥1 locus [32]. Genotyping was repeated for a third time in three cases: 1. the loci that showed discrepancy between the first two PCR assays; 2. the loci that were found homozygous, as it is possible that the genotype represents a true heterozygote where consecutive dropout errors have occurred [33]; and 3. pairs of individuals that differed at only 1 or 2 loci. Consequently, in each locus, the alleles that were accepted were found at least twice for the heterozygous genotypes and three times for the homozygous genotypes. The DNA extracted from NIGS, such as hair, is often at low concentrations and/or highly fragmented [34]. Therefore, due to the total volume of the extracted DNA, it was not possible to perform more than three separate PCR assays in all loci for each sample. Finally, any complete genotype with missing alleles was characterized as unreliable and was excluded from further analysis [35].

### 2.4. Gender Identification

Gender identification was performed using the primers (Table 1) described by Ennis and Gallagher (1994) [30], which amplified the amelogenin gene in male and female chromosomes. PCR amplification was accomplished using the following conditions: initial denaturation at 95 °C for 15 min, 45 cycles of strand denaturation at 94 °C for 30 s, annealing at 60 °C for 30 s, and elongation at 72 °C for 1 min. Final elongation was achieved at 72 °C for 10 min. Amplifications were performed with a reaction volume of 10 μL, containing 1 μL of 10× Reaction Buffer, 0.1 μL of 10× BSA, 0.25 mM dNTPs, 1p/μL of each primer, and approximately 50 ng of template DNA. Moreover, 0.4 units of Taq polymerase and 2 mM MgCl_2_ were used.

Visualization of the PCR products was achieved by electrophoresis in 2% agarose gel. If the sample corresponds to a female brown bear, one band appears on the gel (265 bp); if the sample corresponds to a male bear, two bands appear after electrophoresis (265 bp, 206 bp).

### 2.5. Capillary Electrophoresis

High-resolution capillary electrophoresis was performed using a QIAxcel DNA high resolution kit (Qiagen, Hilden, Germany) on a QIAxcel Advanced System (Qiagen, Hilden, Germany), according to the manufacturer’s instructions. A QX DNA Size Marker (Qiagen) with 10 fragment sizes, ranging in size from 25 to 500 bp, was used to size PCR products. A QX Alignment Marker (Qiagen), which consisted of 15–600 bp fragments, was injected into the cartridge with each sample. The 0 M800 method in the QIAxcel ScreenGel 2.0 software (Qiagen) was used for all analysis; this corresponds to a 10 sec sample injection time at 5 kV and an 800 s separation time at 3 kV. The QIAxcel system injected 0.1 μL of 20 μL PCR products into a cartridge for analysis. The retention time of the PCR fragments relative to the 15 bp and 600 bp QX Alignment Marker fragments was calculated using the ScreenGel software (Qiagen). The PCR product sizes were then determined by comparing the retention time with the QX DNA Size Marker. The ScreenGel software produces a digital gel image and an electropherogram for fragment analysis.

The high detection sensitivity provided by the QIAxcel Advanced System enables robust results even with low concentrations of nucleic acid. QIAxcel Advanced System has a high resolution for fragments smaller than 0.5 kb and ensures great accuracy and confidence in data interpretation. Sample consumption is less than 0.1 μL per analysis, saving precious samples for further downstream analysis.

### 2.6. Statistical Analysis

“Dropout” software [36] was used to determine whether a sample contains genotyping errors and the relative magnitude of the problem as well as the number of unique genotypes.

To evaluate the suitability of the marker set for identifying individuals, the probability of identity (PID) [37] and the more conservative probability of identity among siblings (PID-Sib) [38] were estimated using the software “Gimlet v. 1.3.2” [39].

The observed (H_o_) and expected (H_e_) heterozygosity values for each locus and population were calculated using GENEPOP 4.0. Deviation from Hardy-Weinberg equilibrium was tested using Fisher’s exact tests [40] with unbiased *p*-values derived by a Markov chain method with the same software. The significance value for multiple significance tests was set using the sequential Bonferroni procedure [41]. CERVUS 3.0.3 [42] was used to evaluate polymorphic information content (PIC), null allele probability (F_null_), and number of alleles (A) for each locus.

To test for recent genetic bottlenecks, deviations from expected heterozygosity were inferred under the assumption of mutation drift equilibrium by either the stepwise mutation model (SMM) or the two-phase model (TPM) using the program BOTTLENECK 1.2.02 [43]. The data were analyzed with the recommended settings [44].

The Ne (effective population size) of the brown bear population was estimated using one-point estimate methodologies implemented in NeESTIMATOR 1.3 software [45]. Ne was calculated using the linkage disequilibrium method option of NeESTIMATOR.

The Nc (total population size) was estimated using the estimator implemented in the capture-mark-recapture-based program for non-invasive genetic sampling, CAPWIRE [46]. CAPWIRE accommodates data with multiple observations of an individual within a single session and appears to work well for small populations (<100 individuals), such as the one expected in our study area [46]. Possible capture heterogeneity in our data, due to the collection of genetic samples from power poles [15], necessitated the use of the two-innate rate model (TIRM) for the calculation of population size.

## 3. Results

### 3.1. Individuals and Gender Identification

During the 2022 sampling season, a total of 347 hair samples were collected in the field. However, in the present study, 133 (38%) samples had hair tufts with no roots; therefore, they were not included in the analysis, while the rest of the 214 hair samples contained 2–12 hair roots, and they were used for further analysis. PCR amplification of at least 6 microsatellite loci, as well as a sex marker, was feasible in 136 samples (63%). Amplification of all the 15 microsatellite markers, as well as a sex marker, was accomplished in 83 samples (39%) of the initial 214 hair samples, and they were used for further statistical analysis. From the 1245 genotypes obtained for the 15 loci in hair samples, 10% of the genotypes differed in the alleles between the first and the second PCR, and thus a third PCR reaction was performed.

**Figure 3:**
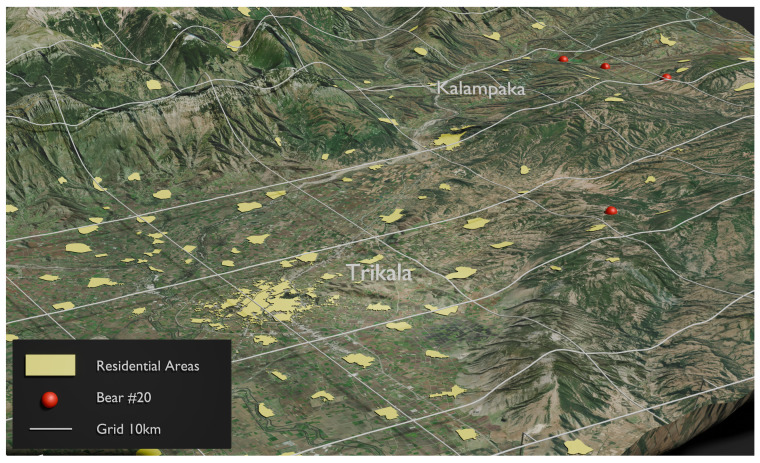
The red dots show the “capture” locations for brown bear No. 20, which was recaptured in both the eastern and western regions of the study area.

**Figure 4:**
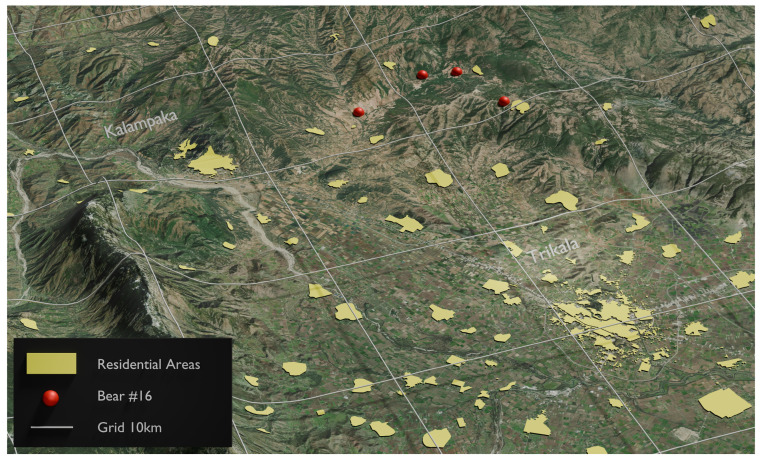
The red dots show the “capture” locations for brown bear No. 16, which was recaptured only in the eastern region of the study area.

From the above 83 samples, 54 unique genotypes were identified, representing 54 different brown bear individuals (Supplementary Material Table S1). Moreover, 33 individuals of the unique bears were “captured” only once, while the rest 21 individuals were “captured” 2–4 times, with the mean arrest/sample ratio being 1.53. Details about the number, the location, the date, and the distance between “recaptures” are given in Supplementary Material Table S2. In most of the cases, the “recaptured” samples were collected at different locations but in relatively short distances (maximum distance 15 km), considering the rugged terrain. Only in two cases of female bears were they “recaptured” 26 km away (Figure 3 and Figure 4). Gender identification was achieved in all 54 unique samples, and most of them (40 individuals) were males (Supplementary Material Table S1). Thus, males were 2.85 times more numerous than females, meaning the sex ratio was about 3 males:1 female.

### 3.2. Genetic Diversity

All loci in the study were polymorphic, with the number of alleles per locus ranging between 4 and 10, with the mean number of alleles being 7.07 (Table 2). Mu59 was the less polymorphic one with an observed heterozygosity value of 38.9%, while G1A, G10C, and REN145P07 were the most polymorphic with an observed heterozygosity value of 94%. In all loci, heterozygotes are more than 40, except for Mu59 (21 heterozygotes and 33 homozygotes). Additionally, Mu59 and, to a lesser degree, Mu23, revealed great deviation between observed and expected heterozygosity values due to the presence of null alleles (F_null_ = 0.3234). More than 85% of the selected markers had high PIC values (mean = 0.765), pointing to the high degree of informativeness of these markers in evaluating genetic diversity. The probability of identity among siblings (P_ID-Sib_) was lower than 0.05 for most of the loci (except for G10H and G10L), indicating a low presence of siblings and recommending that the data can be used for population size estimation [38].

Concerning Hardy–Weinberg tests per locus, almost all loci, except for G10P and CXX, show deviation from Hardy–Weinberg equilibrium with statistically significant *p*-values (*p* ≤ 0.001). Additionally, the population showed deviation from the Hardy–Weinberg equilibrium. The inbreeding coefficient value over all loci was very low (Fis = −0.01), indicating a lack of heterozygosity deficiency and thus a lack of inbreeding. The mean observed heterozygosity was 0.8099, and the unbiased expected heterozygosity was 0.8011.

Tests for bottleneck phenomena were significant for both stepwise and two-mutation phase models for any sample (0.00015 < *p* < 0.0256 and 0.00002 < *p* < 0.0004, respectively), but showed normal L-shaped distributions in mode shift. Thus, the analysis showed evidence of a recent genetic bottleneck.

### 3.3. Estimated Effective (Ne) and Census (Nc) Population Size

In total, 54 individuals were identified according to their composite genotype (Supplementary Material Table S1). Thus, the minimum population size of bears in the Trikala-Meteora project area during 2022 is, according to our study, 54 individuals. Point estimates of Ne using NeESTIMATOR gave a mean value of 99 (95% CI = 73.1–186.7).

As already mentioned, the number of “recaptures” per individual ranged from one to four. Most of the individuals (*n* = 33) were “captured” only once, while 21 bears were “recaptured” once or more. The sex ratio for the individuals that were recaptured was 17 males/4 females.

The analysis with program CAPWIRE, which is based on the number of captures and recaptures, assuming all samples had been collected in a single sampling session, resulted in a point estimation (Nc) of 116 individuals. The 95% confidence interval (CI) was 95–165 individuals. The confidence interval is broad (>50% of point estimation), probably because of the low number of recaptures in relation to the total number of captures.

## 4. Discussion

### 4.1. Reliability of Non-Invasive Genetic Sampling of Biological Material

The reliability of non-invasive genetic sampling largely depends on the quality of the biological material, particularly hair roots, which are essential for successful DNA extraction and PCR amplification of selected microsatellite markers. A 2014 study conducted in the Kastoria region [11] demonstrated that DNA extraction was successful even with samples containing as few as 2–3 high-quality hair roots, emphasizing the significance of hair quality in genetic analysis. This method of non-invasive hair sampling has proven to be an effective way to obtain sufficient genetic data from the brown bear sub-population in the Trikala-Meteora project area without directly handling or disturbing the animals. However, a key challenge with non-invasively collected samples is that they often contain insufficient genetic material, especially when dealing with hair or fecal samples. Furthermore, the DNA extracted from these samples may be degraded or contaminated with PCR inhibitors, which can hinder the amplification of genetic loci. Despite these obstacles, the integrity of hair roots remains a critical factor influencing the success of genetic analysis. In our study, samples containing between 2 and 12 hair roots achieved a 63% amplification success rate, underscoring the importance of obtaining high-quality material. Ensuring a sufficient quantity and quality of biological material not only improves amplification success but also increases the number of genotyped samples, which is crucial for generating more accurate estimates of genetic diversity indices and population sizes.

### 4.2. Genetic Diversity Values of the Population

Several studies have been consulted regarding the genetic diversity of the Greek brown bear population, covering various areas of the Pindos and Peristeri mountain ranges, as well as the Rodopi mountain range [2,12,22,24,35]. To our knowledge, this study is the second one that includes bears from the South Pindos region, near the Trikala-Meteora project area. The only previous study, conducted by Karamanlidis et al. (2018) [22], analyzed samples collected between 2007 and 2010 from the South Pindos area, examining the same 15 microsatellite loci. This allows for direct comparison with the results of the present study. The Trikala-Meteora bear sub-population exhibits high levels of nuclear genetic diversity, with observed and expected heterozygosity values (H_o_ = 0.80 and H_e_ = 0.80) that are slightly higher than those reported by Karamanlidis et al. (2018) [22] for South Pindos (H_e_ = 0.680). These values also surpass those reported for other bear sub-populations in different biogeographical regions of Greece [2,11,12,35] and are comparable to the genetic diversity observed in larger brown bear sub-populations from Northern Europe, Romania, and Russia [47]. The relatively high heterozygosity values in our study suggest a growing population in the region that retains significant levels of genetic diversity. This finding is further supported by the fact that the minimum number of identified individuals in this study is approximately three times higher than the number reported by Karamanlidis et al. [5] thirteen years earlier, confirming positive population trends for the sub-population.

Concerning the brown bear sub-population in the Trikala-Meteora area of Central Greece, the analysis using BOTTLENECK software detected evidence of a recent bottleneck event. In contrast to our findings, isolated and endangered bear populations in Spain and Italy, which have experienced severe historical demographic bottlenecks, exhibit much lower values of observed heterozygosity (H_o_ = 0.28–0.44) and fewer alleles per locus (A = 1.7–3.3) [8,48]. Although detailed information about the demographic history of the bears in the Trikala-Meteora project area is lacking, the observed heterozygosity values indicate that, despite the population undergoing a bottleneck event, it has likely experienced rapid expansion. To obtain a more accurate estimate of the long-term effects of bottleneck events on genetic diversity, future studies should focus on the magnitude and duration of the bottleneck, as well as the rate of genetic recovery. These factors would help describe the population’s ability to adapt to future environmental changes. Additionally, constant gene flow through ecological corridors connecting the project area with neighboring sub-populations across the Pindos mountain range [49] may have contributed to the subsequent recovery of the local bear sub-population.

Moreover, the inbreeding coefficient (Fis) across all loci was found to be very low, aligning with findings from similar studies on other bear sub-populations in Greece [2,11]. This suggests an absence of heterozygosity deficiency and, consequently, a lack of inbreeding. The combination of a low Fis value and high heterozygosity points to a population that is currently in a healthy conservation status, with no immediate risk of genetic depletion in the near future.

### 4.3. Estimated Effectiveness and Census Population Size

One of the main goals of this study was to estimate the size of the brown bear sub-population in the Trikala-Meteora project area. The amplification of 15 microsatellite loci enabled the identification of 54 individual bears, indicating a minimum population size of this magnitude. Notably, most of the identified bears were males, with their numbers nearly three times higher than those of females. To avoid sex bias introduced by the sampling method, it is recommended that sampling be repeated outside the reproductive season to determine whether this bias persists. This would help clarify whether the imbalance is a temporary behavioral phenomenon or a reflection of population structure. Such an approach would enable a more accurate analysis of gender composition and minimize the risk of biased estimates of the population’s genetic diversity indices. This discrepancy may be attributed to differences in capture probabilities and the marking behavior of males compared to females during the mating period. Specifically, rubbing and marking behavior on power poles has been observed to be more pronounced among male bears, resulting in unequal hair sample collection between the sexes [2,11,12,24,50].

The census population size (Nc) was estimated at 116 individuals, which represents a point estimate significantly higher—about two times more numerous—than the minimum population identified (54 individuals). This non-invasive, DNA-based method for estimating Nc can be effectively applied from a single sampling session, making it particularly advantageous for species that are costly or time-consuming to sample. However, we cannot completely rule out the potential violation of one of the model’s key assumptions: population closure [46,51]. The “closed population” assumption posits that there is no immigration, emigration, birth, or death within the population during the sampling period. While we know that no births occurred during our sampling period—since bears typically give birth in late winter—we cannot exclude the possibility of bear deaths, potentially due to poaching. Additionally, based on the observed heterozygosity values, the Trikala-Meteora bear sub-population appears to be growing. However, movements in and out of the sampling area over the years could negatively affect recapture probabilities. In such cases, estimates derived from closed-model methods likely reflect a larger population than just the animals within the predefined sampling area, encompassing also the surrounding regions [52].

A study conducted in the Rodopi mountain range (eastern population nucleus) [2] estimated the census population size at 91 individuals (95% CI: 41–262), while the effective population size was calculated at 42.2 (95% CI: 25.3–97.7). This discrepancy can be attributed to the constant transboundary movements of individuals between Greece and Bulgaria. Similar findings, where the census population size is two to three times higher than the minimum number of identified individuals, have also been reported in other studies across Greece [11,12]. These results suggest that the CAPWIRE estimate corresponds to a larger population inhabiting an area that extends beyond the immediate study site. Moreover, it is essential to consider that, as recommended by Miller et al. (2005) [46], recapture rates should ideally be between 2 and 3 observations per individual. In our study, the recapture rate was only 1.53 observations per individual, which is significantly lower than the recommended threshold. This limitation indicates that the estimates should be interpreted with caution. To achieve more accurate population size estimates, intensive sampling efforts aimed at increasing the recapture ratio will be necessary. The effective population size (Ne) was estimated at 99 (95% CI: 73.1–186.7), which is substantially higher than the minimum number of 54 individuals considered sufficient to mitigate inbreeding issues within the population [53]. High Ne estimates have also been reported by Karamanlidis et al. [22] for the South Pindos area, suggesting that the Ne estimate may actually apply to the broader Pindos region rather than being confined to individual sub-populations.

Long-term monitoring of Ne is recommended, as high Ne values over time reduce the likelihood of deleterious genetic drift. Such an approach could provide a more robust understanding of the sub-population’s genetic resilience in response to environmental pressures and future demographic changes. Overall, the high Ne estimate, combined with elevated heterozygosity values and low Fis detected in the present study, indicates a population experiencing growth and expansion beyond the studied area. In a growing population, the variance effective population size tends to increase, further supporting the notion of a robust and expanding bear population in this region [54].

### 4.4. Existing Corridor and Gene Flow from West to East (Or Vice Versa) in the Studied Sub-Areas

**Figure 5:**
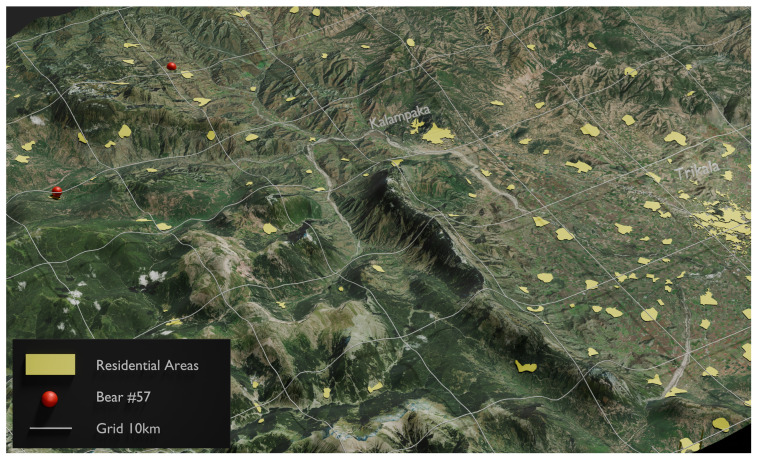
The red dots show the “capture” locations for brown bear No. 57, which was recaptured in both the eastern and western regions of the study area.

Another notable outcome from the present study was the movement of individuals between the western and eastern parts of the studied sub-areas. Specifically, two individuals, identified as No. 20 (Figure 3) and No. 57 (Figure 5) (Supplementary Material Table S2), were recaptured in both the eastern and western regions of the study area. This observation provides the first evidence of individuals migrating from the western, most robust, and permanent bear sub-population to the eastern portion of the study area, which has been recolonized over the past decade. Alternatively, this movement could also indicate bears traveling from the east to the west, utilizing an existing natural corridor that allows them to navigate around various artificial and human-related barriers, such as the new E65 highway currently under construction.

Given the potential impact of this highway on bear movement, it is crucial to further investigate and monitor the effects of this barrier on the functionality of the corridor connecting the eastern and western parts of the study area. Understanding how such infrastructure affects wildlife movement will be essential for effective conservation planning and ensuring the long-term viability of bear sub-populations in the region.

## 5. Conclusions

One of the primary threats to biodiversity is habitat fragmentation [55]. As ecosystems become increasingly fragmented, populations become disconnected from one another, leading to significant challenges for species survival. Fragmentation can restrict the movement of organisms, isolating them in smaller areas and reducing their access to resources, mates, and genetic diversity. Simultaneously, effective dispersal is a critical mechanism by which organisms expand their ranges and move between populations [49]. This dispersal not only facilitates colonization of new habitats but also maintains genetic connectivity among sub-populations, which is essential for ensuring long-term population viability [56]. Although the violation of some statistical parameters underlines the need to treat estimates cautiously, the results of the present study support the hypothesis of a growing population that remains in constant contact with neighboring sub-populations in the Pindos Mountain range. This sub-population appears to be in good conservation status and does not seem to be threatened by genetic erosion in the forthcoming years. However, it is crucial to prioritize long-term monitoring and management actions aimed at maintaining sufficient levels of gene flow between sub-populations. Furthermore, it is important to note that estimating population size based on a single sampling session provides only a snapshot of the population’s dynamics at that specific time. To obtain more accurate population estimates, it is necessary to conduct multiple consecutive sampling sessions. This approach will help increase the number of high-quality samples and improve the reliability of the data collected. Additionally, incorporating scat sampling in future surveys will be beneficial in addressing potential underestimations of female bear populations, as it can provide valuable genetic material for analysis.

## Figures and Tables

**Table 1:** Primer sequence and size range of the 15 microsatellite loci (T_a_: annealing temperature).

Primer Name	Sequence	Size Range (bp)	T_a_ (°C)
G10H F [25]	5′-ATCCAACAAGAAGACCACTGTAA-3′	221–273	58
G10H R	5′-GATCAGAGACCACCAAGTAGG-3′		
G10L F [26]	5′-GAAGATACAGAAACCTACCCATGC-3′	120–174	58
G10L R	5′-GTACTGATTTAATTCACATTTCCC-3′		
Mu50 F [27]	5′-GGAGGCGTTCTTTCAGTTGGT-3′	106–146	54
Mu50 R	5′-TGGAACAAAACTTAACACAAATG-3′		
G10P F [26]	5′-AGGAGGAAGAAAGATGGAAAAC-3′	135–165	54
G10P R	5′-CATAGGAGGAAGAAAGATGGAAA-3′		
Mu59 F [6]	5′-GCTGCTTTGGGACATTGTAA-3′	217–255	58
Mu59 R	5′-CAATCAGGCATGGGGAAGAA-3′		
G10C F [26]	5′-AAAGCAGAAGGCCTTGATTTCCTG-3′	89–117	58
G10C R	5′-GGGGACATAAACACCGAGACAGC-3′		
G1D F [26]	5′-ATCTGTGGGTTTATAGGTTACATCAA-3′	202–220	54
G1D R	5′-CTTGATACCTAGCACCCAGCAAGG-3′		
G1A F [26]	5′-ACAGTCGACCCTGCATACTCTCCTCTGATG-3′	174–202	54
G1A R	5′-GCACTGTCCTTGCGTAGAAGTGAC-3′		
G10J F [25]	5′-GATCAGATATTTTCAGCTTT-3′	73–112	52
G10J R	5′-AACCCCTCACACTCCACTTC-3′		
Mu23 F [27]	5′-TCCCCAGCGGATGGATG-3′	85–113	58
Mu23 R	5′-CCCAATGGGTTTCTTGTTT-3′		
Mu51 F [6]	5′-CAGCCAGAATCCTAAGAGACC-3′	94–124	54
Mu51 R	5′-AGGGACAGGAGGTAGTTGCT-3′		
G10M F [6]	5′-TTCCCCTCATCGTAGGTTGTA-3′	174–204	54
G10M R	5′-AATAATTTAAGTGCATCCCAGG-3′		
CXX110 F [28]	5′-AATCTAAGCCAATATTCTCC-3′	101–135	40
CXX110 R	5′-GCATCCAAGTAAATCAAGA-3′		
REN145P07 F [28]	5′-TGGAAAGGTTTGCACTCTGA-3′	154–182	54
REN145P07 R	5′-AGCCTCCCCATTTCACAGAT-3′		
G10U F [29]	5′-TGCAGTGTCAGTTGTTAGGAA-3′	145–185	54
G10U R	5′-GTATTTCCAATGCCCTAAGTGAT-3′		
AMELO F [30]	5′-TGACTCCAACCCAACACCAC-3′	206, 265	60
AMELO R	5′-CCCGCTTGGTCTTGTCTGTTGC-3′		

**Table 2:** Details of the studied microsatellite loci *.

Locus	A	R	H_o_	H_e_	PIC	PHW	F_null_	Fis	P_ID_	P_ID-Sib_
G10H	7	54	0.778	0.819	0.785	0.0000	0.0129	0.0507	6.18 × 10^−2^	3.60 × 10^−1^
G10L	9	54	0.852	0.819	0.792	0.0005	−0.0242	−0.0403	3.38 × 10^−3^	1.29 × 10^−1^
MU59	7	54	0.389	0.772	0.73	0.0000	0.3234	0.4989	3.05 × 10^−4^	5.02 × 10^−2^
G1A	7	54	0.944	0.825	0.793	0.0000	−0.0803	−0.1458	1.76 × 10^−5^	1.79 × 10^−2^
G10C	8	54	0.944	0.823	0.791	0.0462	−0.0795	−0.1497	1.03 × 10^−6^	6.38 × 10^−3^
G1D	7	54	0.741	0.767	0.729	0.0226	0.0189	0.0342	9.06 × 10^−8^	2.50 × 10^−3^
MU50	7	54	0.907	0.807	0.771	0.0000	−0.0688	−0.1255	6.23 × 10^−9^	9.18 × 10^−4^
Mu51	5	54	0.907	0.758	0.712	0.000	−0.1155	−0.199	6.27 × 10^−10^	3.67 × 10^−4^
G10M	4	54	0.833	0.71	0.644	0.0384	−0.0892	−0.1757	9.23 × 10^−11^	1.60 × 10^−4^
G10J	6	54	0.796	0.785	0.742	0.000	−0.0196	−0.0147	7.84 × 10^−12^	6.11 × 10^−5^
G10U	7	54	0.833	0.801	0.767	0.0217	−0.0257	−0.0406	5.42 × 10^−13^	2.26 × 10^−5^
REN145	7	54	0.944	0.838	0.809	0.000	−0.0685	−0.1281	2.72 × 10^−14^	7.86 × 10^−6^
Mu23	10	54	0.667	0.885	0.865	0.000	0.1371	0.2487	7.47 × 10^−16^	2.50 × 10^−6^
CXX	6	54	0.741	0.745	0.706	0.5772	0.0124	0.0052	7.48 × 10^−17^	1.02 × 10^−6^
G10P	9	54	0.87	0.861	0.837	0.1664	−0.0147	−0.0105	2.81 × 10^−18^	3.38 × 10^−7^
Μean	7.067		0.8099	0.8011	0.765	0.000		−0.0111		

* Number of alleles (A), allelic size range in base pairs (R), expected and observed heterozygosity (H_e_, H_o_), polymorphic information content (PIC), probability value for Hardy-Weinberg tests (PHW), null alleles per locus (F_null_), inbreeding coefficient value (Fis), probability of identity (P_ID_), and the probability of identity among siblings (P_ID-Sib_) for the Trikala-Meteora brown bear sub-population.

## Data Availability

Data of this study is presented within the manuscript and the Supplementary Materials.

## Supplementary Materials

The following supporting information can be downloaded at: https://www.mdpi.com/article/10.3390/ani14233530/s1, Table S1: 54 genotypes representing 54 different bears and their gender as revealed from the analysis of 83 samples (*n* = 83). Table S2: The 21 “recaptured” bears and the maximum distance between recaptures.
