# Supplementary material for: Genetic Analysis of the Brown Bear Sub-Population in the Pindos Mountain, Central Greece: Insights into Population Status and Conservation
Source: Animals (Basel). 2024 Dec 6;14(23):3530. doi: 10.3390/ani14233530 (PMC11640096; doi:10.3390/ani14233530)
Supplement: Supplementary file 1 [file animals-14-03530-s001.zip › animals-3313368-supplementary.pdf]

Supplementary Material

Table S1. *54 genotypes representing 54 different bears and their gender as revealed from the analysis of 83 samples (n=83).*

	G10H		G10L		MU59		G1A		G10C		G1D		MU50		Mu51		G10M		G10J		G10U		REN145		Mu23		CXX		G10P		Sex
1	223	255	141	159	225	227	186	190	201	205	178	182	128	132	206	216	204	208	178	188	177	179	155	165	185	185	147	153	163	167	Male
4	223	255	155	157	227	227	188	190	199	205	178	184	128	132	206	216	208	208	180	188	171	177	155	167	195	195	147	155	149	165	Male
6	233	255	141	145	225	225	188	190	205	209	182	188	130	132	206	210	204	204	184	190	171	173	155	159	195	195	147	147	149	161	Male
8	223	255	141	157	221	229	190	194	203	205	182	186	130	132	210	214	204	208	184	188	169	169	155	159	197	201	153	155	149	159	Male
10	231	239	141	157	219	227	184	186	195	203	182	184	128	132	214	216	204	208	180	188	169	171	159	163	201	201	153	157	147	159	Male
11	223	255	155	157	221	227	190	194	203	205	182	188	130	136	206	216	206	206	180	188	169	169	159	163	197	201	155	157	159	159	Male
12	253	253	153	157	227	239	186	190	199	201	182	184	130	134	206	216	204	208	186	186	175	179	159	165	195	207	149	155	153	159	Male
13	223	223	141	153	219	227	186	188	201	205	182	182	126	130	206	216	204	208	180	188	169	177	161	163	197	201	147	147	153	161	Male
15	223	255	141	147	227	227	188	192	205	209	180	186	126	130	206	216	206	208	178	186	169	175	155	165	185	185	147	153	149	161	Male
16	233	255	155	159	225	227	192	194	203	207	180	182	128	130	206	218	206	206	180	180	169	173	155	167	185	185	147	151	153	163	Male
17	223	255	155	157	221	225	190	194	195	205	182	186	126	130	206	210	200	200	190	190	169	173	155	157	195	199	147	147	161	161	Male
18	231	255	155	157	221	227	186	190	201	203	178	178	130	132	206	210	206	208	180	180	169	171	155	165	195	199	147	155	161	167	Male
20	253	253	141	157	219	227	186	194	195	203	182	182	124	126	206	216	206	208	180	188	169	169	155	167	197	199	147	149	157	157	Male
21	223	231	155	157	219	219	190	194	201	203	184	184	126	130	206	216	204	206	180	188	169	173	155	159	199	205	147	147	159	167	Male
22	253	253	153	157	219	219	188	190	205	207	180	184	132	132	214	214	204	206	180	188	169	173	161	165	197	197	147	153	147	153	Male
23	231	255	159	159	227	227	180	180	201	201	182	182	132	132	206	210	200	204	180	188	175	179	165	167	199	207	147	153	161	161	Male
24	233	255	157	157	225	227	184	186	201	203	178	182	124	126	206	210	204	206	180	188	173	175	165	167	193	199	147	155	159	161	Male
27	231	255	155	157	225	225	188	192	199	201	176	182	128	130	206	210	200	204	180	188	173	175	163	165	199	207	149	155	159	163	Male
30	223	255	141	157	225	241	188	192	197	201	178	182	124	126	206	216	204	208	180	188	169	175	167	167	193	207	151	157	149	159	Male

32	231	231	141	145	225	225	190	194	205	209	182	182	130	136	206	210	200	204	184	188	171	177	157	159	191	195	149	151	157	161	Male
33	231	253	143	147	225	225	186	190	203	205	180	184	128	132	206	216	204	206	178	186	169	177	155	167	193	207	147	155	153	161	Male
35	253	253	153	159	227	227	186	190	201	207	178	184	128	132	206	210	200	206	178	188	159	171	155	165	191	201	153	157	161	165	Male
36	223	233	153	157	225	225	186	194	201	205	178	184	128	130	206	210	204	206	186	186	171	177	161	165	193	205	147	151	149	157	Male
38	233	239	153	157	225	225	186	188	203	205	178	184	128	130	206	216	204	206	178	188	171	175	155	167	193	197	149	153	153	159	Male
39	233	255	155	157	225	225	188	192	199	209	182	188	130	132	206	210	204	206	178	186	171	175	163	167	195	207	147	157	157	159	Male
40	223	223	157	161	225	241	186	190	201	205	176	182	132	136	206	214	206	208	178	178	169	177	155	159	191	195	147	155	161	167	Male
41	223	233	159	161	219	219	190	194	199	203	180	184	128	130	206	218	206	208	178	186	169	171	155	157	195	195	147	149	159	167	Male
42	233	255	157	159	225	225	184	186	201	207	178	184	128	132	206	210	204	206	178	186	171	175	155	165	193	205	147	157	149	159	Male
44	255	255	153	159	219	219	190	194	201	203	178	182	128	130	214	214	206	208	180	186	169	169	161	165	195	195	147	157	147	149	Male
45	233	241	155	157	227	227	184	186	195	203	182	182	128	132	216	218	204	206	178	180	169	171	159	161	193	193	153	157	147	157	Male
47	231	239	157	161	221	227	184	186	195	203	180	184	126	130	214	216	204	208	178	186	171	173	161	165	199	205	147	153	147	157	Male
49	233	233	159	161	221	221	188	190	197	205	182	182	128	130	214	214	206	208	178	186	169	169	163	167	195	195	147	147	149	157	Male
50	233	255	157	159	241	241	190	192	205	205	176	178	132	136	210	214	206	208	180	184	177	179	155	165	193	205	153	155	157	161	Male
51	231	233	157	159	219	227	184	186	197	205	182	186	130	134	214	214	204	208	180	188	169	171	161	165	199	205	147	153	159	167	Male
52	233	255	143	147	239	239	190	194	201	205	180	186	128	130	206	210	204	206	180	180	159	173	157	157	195	199	147	147	149	159	Male
54	223	255	157	157	227	227	188	192	199	205	178	182	132	136	206	218	206	208	178	186	169	175	155	167	193	193	147	155	149	159	Male
55	233	255	141	145	225	227	188	192	203	205	178	182	128	132	206	218	204	206	178	186	169	175	157	163	197	203	147	147	147	153	Male
56	223	255	155	159	221	227	190	194	201	203	182	182	126	136	206	216	204	208	180	188	169	171	163	167	193	197	153	155	149	159	Male
57	233	241	143	145	229	229	190	194	203	205	182	182	128	132	206	218	204	206	180	186	169	175	157	159	199	199	147	155	153	167	Male
60	231	233	159	159	221	221	190	194	201	205	182	186	128	132	206	216	206	208	180	188	171	171	155	157	195	195	147	147	147	165	Male
1	233	241	157	157	227	227	190	192	207	207	182	188	124	124	206	210	204	204	180	188	171	173	155	155	195	199	147	147	163	163	Female
2	233	255	141	145	225	225	186	190	203	207	184	184	128	130	206	214	208	208	180	188	171	173	161	165	197	197	149	157	149	153	Female
3	233	255	155	157	225	225	190	194	205	207	182	188	128	130	210	218	204	206	188	190	169	177	161	165	201	207	151	153	159	161	Female
5	231	253	141	155	225	227	188	194	203	205	180	182	130	132	206	216	206	208	180	188	169	175	155	159	195	199	149	151	159	163	Female

7	223	231	155	157	227	227	188	192	199	205	178	182	126	130	206	216	206	208	180	190	169	175	155	159	191	191	155	157	159	167	Female
9	233	233	157	157	225	225	188	190	201	205	184	184	126	130	206	210	206	206	178	180	159	173	155	159	201	205	155	155	159	161	Female
10	253	253	157	157	219	239	190	194	197	205	180	184	126	130	206	216	204	208	186	186	169	173	155	159	191	193	147	147	159	161	Female
11	233	241	141	159	225	225	190	194	201	205	178	184	128	134	214	216	204	208	178	178	159	159	155	159	191	203	147	155	159	167	Female
12	233	233	157	157	221	221	188	188	195	203	182	182	126	130	216	216	206	208	178	178	169	169	159	161	193	193	147	147	159	165	Female
13	233	241	157	161	227	227	184	186	195	203	184	188	132	132	214	216	204	208	178	186	171	173	161	165	197	203	153	157	147	147	Female
14	231	233	143	147	239	239	190	194	201	203	180	184	132	134	206	218	200	204	178	178	159	159	155	157	199	199	147	147	149	159	Female
15	231	233	143	147	221	227	184	188	203	207	180	184	132	132	206	216	206	208	180	188	169	175	155	157	205	207	153	155	149	161	Female
16	223	255	143	157	225	225	180	180	199	205	182	182	130	134	206	210	206	208	180	188	169	173	155	165	197	205	147	147	161	161	Female
20	233	239	157	159	227	227	188	190	195	203	182	186	130	134	214	216	204	204	180	188	169	171	165	167	199	203	151	155	159	167	Female

__Table S2.__ The 21 "recaptured" bears and the maximum distance between recaptures.

Bear	Area	1st capture	2nd capture	3rd capture	4th capture	Gender
1	Malakasi	29/5/22	29/5/22<br>477m	29/5/22<br>497m	-	Male
4	Panagia	29/5/22	3/6/22<br>2km	-	-	Male
6	Dipotamia	3/6/22	3/6/22<br>0m	-	-	Male
8	Agnantia	4/6/22	4/6/22<br>0m	-	-	Male
13	Matoneri	29/5/22	29/5/22<br>482m	-	-	Male
18	Amarantos	1/6/22	2/6/22<br>14km	-	-	Male
24		6-9/6/2022	6-9/6/2022<br>1.7km	6-9/06/2022<br>7.5km	-	Male
27		6-9/6/2022	6-9/6/2022<br>0m	6-9/6/2022<br>2.8km	-	Male
30	Malakasi	6/7/22	6/7/22<br>176m	-	-	Male
33	Agnantia	9/7/22	9/7/22<br>1.2km	-	-	Male
36	Matoneri	6/7/22	6/7/22<br>482m	-	-	Male
41	Klinovos	8/7/22	8/7/22<br>11.1km	-	-	Male
45		3-6/07/2022	3-6/07/2022<br>1.7km	-	-	Male
47		3-6/07/2022	3-6/07/2022<br>4.2km	-		Male

52		8-10/08/2022	8-10/08/2022<br>751m	-	-	Male
57		5/8/22	5/8/22<br>0m	5/8/22<br>15km	-	Male
60		5/8/22	5/8/22<br>0km	-	-	Male
5	Malaksi	6/7/22	6/7/22<br>1km	-	-	Female
7	Koutsoufliani	9/7/22	9/7/22<br>1.7km	-	-	Female
16		3-6/7/2022	3-6/7/2022<br>898m	3-6/7/2022<br>1.9km	3-6/7/2022<br>26km	Female
20		3-6/7/2023	3-6/7/2023<br>6.7km	3-6/7/2023<br>22.3km	3-6/7/2023<br>23.4km	Female
